# A review on the advances and challenges of immunotherapy for head and neck cancer

**DOI:** 10.1186/s12935-021-02024-5

**Published:** 2021-07-31

**Authors:** Gang Cheng, Hui Dong, Chen Yang, Yang Liu, Yi Wu, Lifen Zhu, Xiangmin Tong, Shibing Wang

**Affiliations:** 1 Department of Stomatology, Zhejiang Provincial People’s Hospital, People’s Hospital of Hangzhou Medical College, Hangzhou, 310014 China; 2 Department of Stomatology, Bengbu Medical College, 2600 Donghai Avenue, Bengbu, 233030 China; 3 Department of Ultrasonography, Zhejiang Provincial People’s Hospital, People’s Hospital of Hangzhou Medical College, Hangzhou, 310014 China; 4 Phase I Clinical Research Center, Zhejiang Provincial People’s Hospital, People’s Hospital of Hangzhou Medical College, Hangzhou, 310014 China; 5 Key Laboratory of Tumor Molecular Diagnosis and Individualized Medicine of Zhejiang Province, Zhejiang Provincial People’s Hospital, Hangzhou, 310014 Zhejiang People’s Republic of China; 6 Molecular Diagnosis Laboratory, Zhejiang Provincial People’s Hospital, People’s Hospital of Hangzhou Medical College, Hangzhou, China

**Keywords:** Head and neck cancer, Oncolytic viruses, Monoclonal antibodies, Chimeric antigen receptor T cells, Therapeutic vaccines

## Abstract

Head and neck cancer (HNC), which includes lip and oral cavity, larynx, nasopharynx, oropharynx, and hypopharynx malignancies, is one of the most common cancers worldwide. Due to the interaction of tumor cells with immune cells in the tumor microenvironment, immunotherapy of HNCs, along with traditional treatments such as chemotherapy, radiotherapy, and surgery, has attracted much attention. Four main immunotherapy strategies in HNCs have been developed, including oncolytic viruses, monoclonal antibodies, chimeric antigen receptor T cells (CAR-T cells), and therapeutic vaccines. Oncorine (H101), an approved oncolytic adenovirus in China, is the pioneer of immunotherapy for the treatment of HNCs. Pembrolizumab and nivolumab are mAbs against PD-L1 that have been approved for recurrent and metastatic HNC patients. To date, several clinical trials using immunotherapy agents and their combination are under investigation. In this review, we summarize current the interaction of tumor cells with immune cells in the tumor microenvironment of HNCs, the main strategies that have been applied for immunotherapy of HNCs, obstacles that hinder the success of immunotherapies in patients with HNCs, as well as solutions for overcoming the challenges to enhance the response of HNCs to immunotherapies.

## Background

According to GLOBOCAN 2020, head and neck cancers (HNCs), including lip and oral cavity, larynx, nasopharynx, oropharynx, and hypopharynx, account for about 800,000 new cancer cases and 450,000 deaths worldwide [1]. Head and neck squamous cell carcinomas (HNSCCs) that stem from the mucosal epithelium are the most common malignancies in the head and neck. HNSCC is a multifactorial malignancy resulting from infection with high-risk human papillomaviruses (HPVs) and risk factors associated with lifestyles, such as alcohol consumption and smoking [2, 3]. Despite massive improvements in HNSCCs treatment strategies, including surgery, radiotherapy, and chemotherapy, the 5-year overall survival of HNSCC is 30–65%, depending on the access to health resources and systems [4]. A research wave surrounding cancer therapy has resulted in developing novel therapeutic strategies for fighting against cancer in the past two decades.

Cancer immunotherapy is based on modifying the host immune system to induce anti-tumor immune responses and avoid immune escape. There is some biological rationale in the development of immunotherapy in HNCs, especially HNSCCs. First, there is growing evidence that HNSCCs are extremely immunosuppressive malignancy owing to disruption of T-cells signaling, induction of immune tolerance, and immune evasion [5, 6]. Second, infiltration of immunosuppressive cells, including regulatory T (Treg) cells, as well as an increase in the expression of co-inhibitory receptors on T-cells, including programmed cell death protein 1 (PD-1) and cytotoxic T-lymphocyte-associated protein 4 (CTLA-4) in the HNSCCs microenvironment are attractive targets for immunotherapy [7, 8]. Third, the HPVs-associated sub-set of HNSCC has a distinct immune cell profile in the tumor microenvironment (TME), providing a suitable antigenic target [9]. Here, we summarize the immune responses in HNCs, various immunotherapy strategies against HNC, including oncolytic immunotherapy, monoclonal antibodies, chimeric antigen receptor T cells (CAR-T cells), vaccines, and each strategy's challenges.

## Immune response in head and neck cancer

According to the tumor immune surveillance theory, the immune system can identify (pre)cancerous cells and destroy them before developing into detectable and dangerous tumors [10]. However, immune reactions against tumor cells may fail due to the generation of inhibitory immune cells and the release of suppressive cytokines and mediators, leading to immune escape [11]. Thus, the interaction between immune cells within TME with tumor cells shapes tumor cells' behavior and their response to therapeutic agents.

**Fig. 1:**
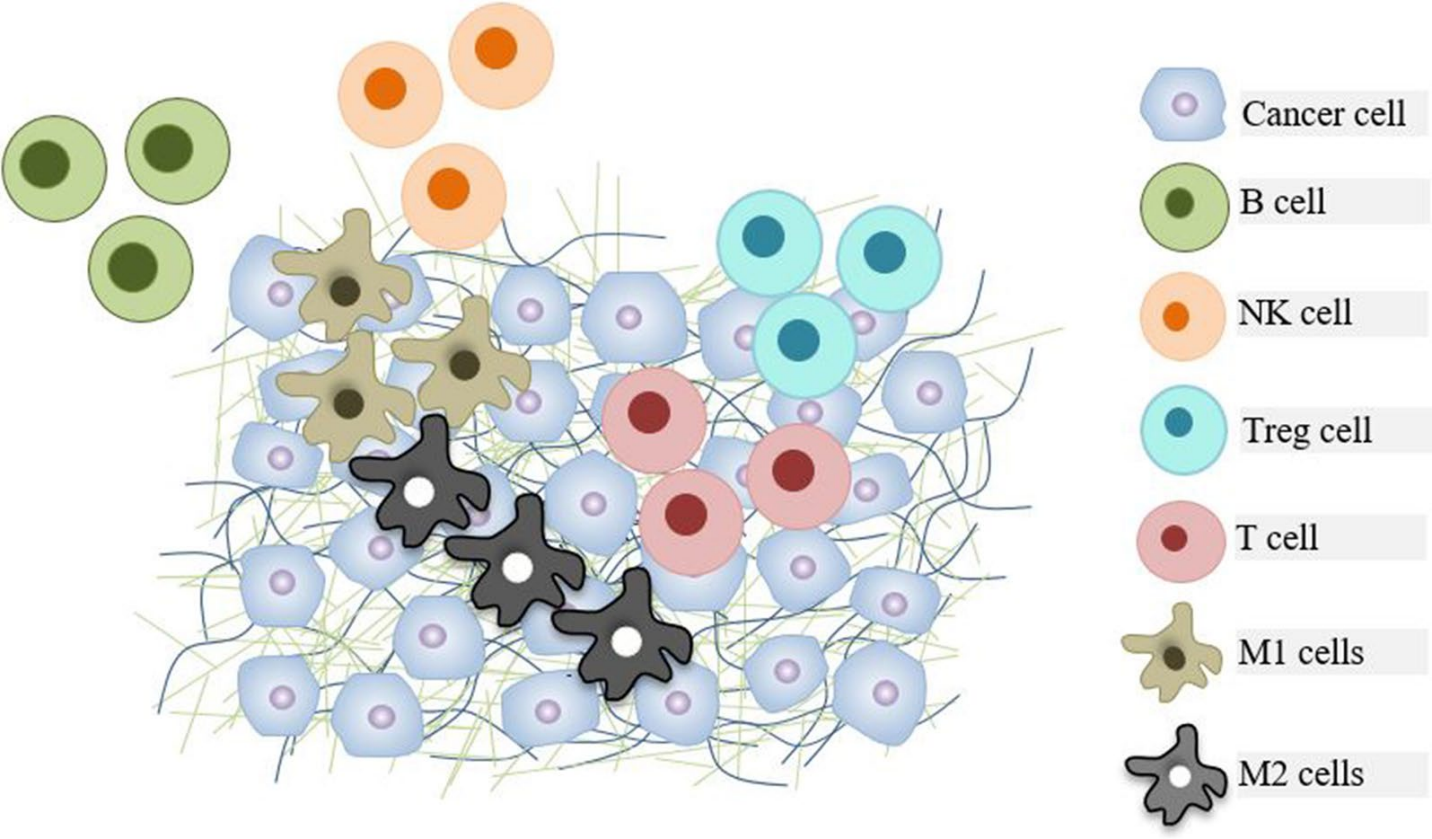
Schematic overview of the tumor microenvironment (TME) and infiltrating immune cells into the TME. Both innate and acquired immune cells infiltrate into the TME and interact with tumor cells, contributing to immunostimulation or immunosuppression

It has been reported that the TME of HNSCC consists of heterogeneous cellular and molecular components, which are associated with a good/poor prognosis in HNSCC patients (Fig. 1). The presence of inhibitory cells, including Tregs and myeloid-derived suppressor cells (MDSCs), as well as tumor-associated macrophages, can promote cancer progression and immune escape. For instance, Jie et al*.* found that intratumoral FOXP3^+^ Tregs create immunosuppressive TME in HNSCC patients by highly expressing immune checkpoint receptors [12]. Shang et al*.* conducted a meta-analysis study encompassing 15,512 patients with 17 types of cancer for analyzing the effect of FOXP3^+^ Tregs on overall survival (OS). They indicated that high infiltration of Tregs was remarkably associated with shorter OS in most of the solid tumors, including melanomas, renal, cervical, and breast cancers, whereas they noticed contrary results in colorectal, oesophageal, and head and neck cancers [13]. Similarly, Seminerio et al*.* showed that higher infiltration of FOXP3^+^ Tregs was associated with longer patients survival [7]. Thus, tumor histological grade, tumor stage, and tumor site are determining factors in the effect of Tregs on patients' survival. Recently, Pang et al*.* reported that the MDSCs number was increased in the tissue of oral squamous cell carcinoma (OSCC) patients, which was positively associated with lymph node metastasis, pathological grade, T stage, and poor prognosis [14]. In a prospective cohort study, Kim et al*.* found that MDSCs were accumulated in peripheral blood of HNSCC patients, specifically in HPV-associated ones, and higher levels of MDSCs were related to advanced cancer stage, metastasis, and poor clinical outcomes [15]. Other immune cells that participate in HNSCC and have adverse effects on clinical outcomes are tumor-associated macrophages (TAMs). The infiltrating macrophages into the TME, named TAMs, can be classified into two classes: M1 (classically activated) phenotype with anti-tumor effects and M2 (alternatively activated) phenotype with pro-tumor effects [16]. M1-like TAMs that express CD40, CD80, and CD86 markers are induced by tumor necrosis factor α (TNF-α), interferon-γ (IFN-γ), and granulocyte–macrophage colony-stimulating factor (GM-CSF), whereas M2-like TAMs which are induced by transforming growth factor (TGF)-β and IL-10, are characterized with the expression of CD163, CD204, and CD206 markers [17]. Moreover, CD86 is a pan marker for macrophage polarization and is expressed on both macrophage phenotypes [18]. A meta-analysis study revealed a higher density of TAMs and M2-like subset macrophages in the TME of HNSCC patients and their association with vascular and lymphatic invasion, nodal involvement, and advanced T stage [19]. Fu et al*.* demonstrated that M2 subtypes could induce radioresistance in HPV‑negative HNSCC by secreting human heparin‑binding epidermal growth factor (HB‑EGF). HB‑EGF can promote the non‑homologous end‑joining (NHEJ) pathway via activating EGFR [20]. Moreover, TAMs are correlated with cancer stem cells and poor prognosis in OSCC patients [21]. On the other hand, M1 macrophages have positive effects on the inhibition of HNSCC. For instance, miR-9-carrying exosomes derived from HPV-associated HNSCC cells can mediate the polarization of macrophages toward the M1 subtypes, leading to an increase in tumor radiosensitivity [22]. Similarly, Chen et al*.* demonstrated M1 macrophages in enhancing radiosensitivity of HPV-positive HNSCC cells [23]. Furthermore, Th1 and Th17 cells contribute to HNCs development. For instance, Costa et al*.* found that HNC development at the premalignant stage is associated with an increase in Th1 and Th17 [24]. Another study also demonstrated that the increase of Th17 cells powerfully correlated with HNSCC metastasis [25]. Th17 cells promote tumor growth by producing IL-17. IL-17 stimulated the production of IL-6 via tumor cells and tumor-infiltrating immune cells, leading to the activation of the signal transducer and activator of transcription 3 (Stat3) pathway and subsequently tumor growth [26]. IL-17 is also involved in the suppression of immune responses against tumor cells by facilitating the infiltration of Tregs and MDSCs into the TME [27]. The increase of IL-17 during the progression of HNSCC undergo a slight decrease in the latest phases due to the higher levels of TGFβ that promotes Treg differentiation while inhibit the differentiation of Th17 cells [28].

## Immunotherapy strategies for head and neck cancer

Cancer immunotherapy aims to eliminate tumor cells or control tumor growth and progression by reinforcing immunosurveillance, increasing the immune effector cells' cytolytic activity, and minimizing immune escape. For these ends, several immunotherapy strategies have been developed for HNSCC during the last two decades. Here, we reviewed various immunotherapy strategies for head and neck cancer and the challenges of each system.

## Oncolytic virotherapy

### Overview of oncolytic virotherapy

**Fig. 2:**
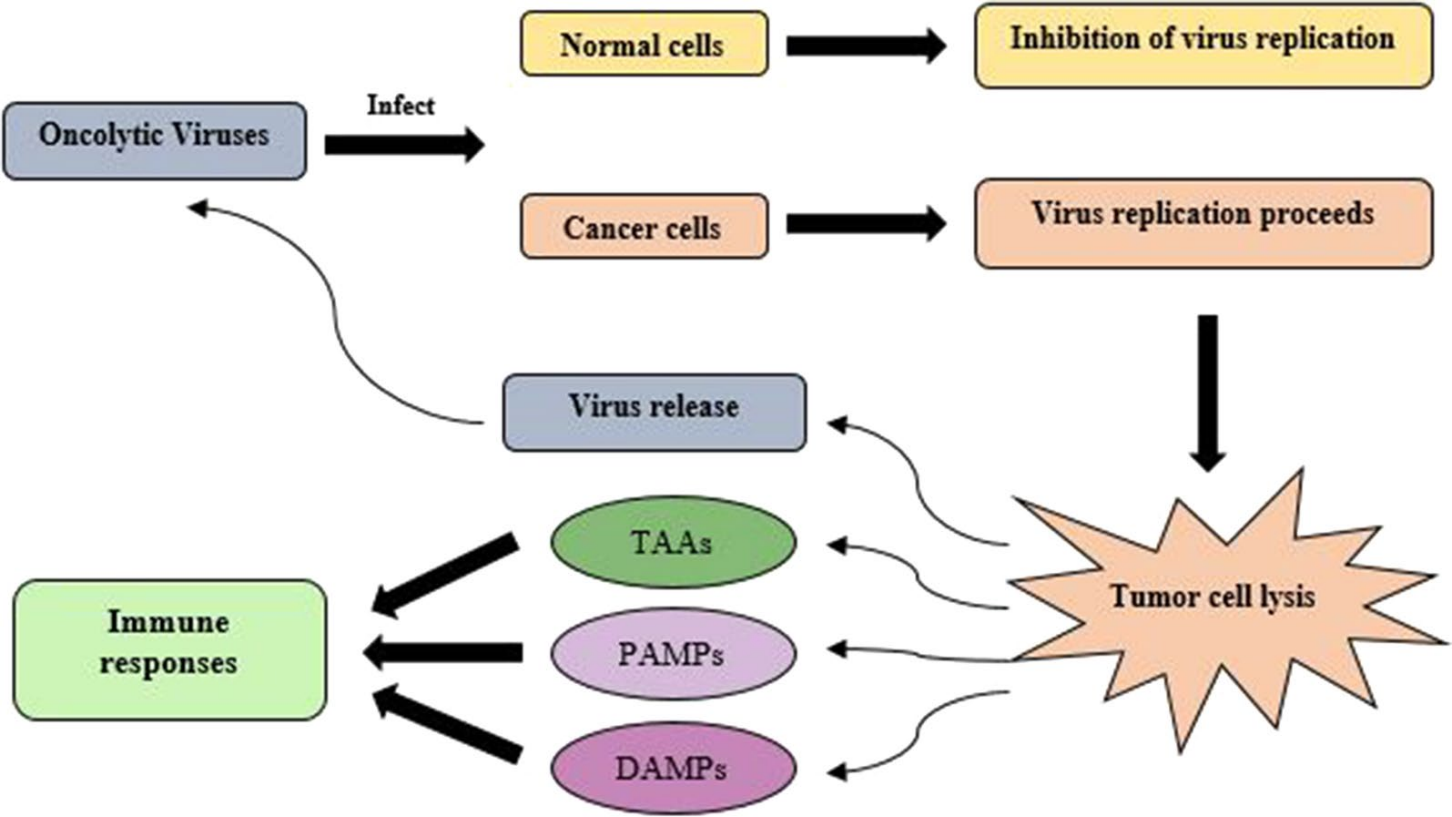
The mechanisms of action of oncolytic viruses. Oncolytic viruses can infect, lyse, and kill the tumor cells, without affecting normal cells. The lysed tumor cells stimulate immune cells by releasing tumor-associated antigens (TAAs), pathogen-associated molecular patterns (PAMPs), and damage-associated molecular patterns (DAMPs). The released virions from the lysed cells can infect other tumor cells and help to destroy the remaining cells

Oncolytic viruses (OVs) can specifically target and cause lysis of tumor cells without infecting normal cells. OVs exert their anti-tumor effects by two mechanisms: directly killing tumor cells and indirectly augmentation of anti-tumor immune responses via releasing pathogen-associated molecular patterns (PAMPs) and damage-associated molecular patterns (DAMPs) as well as tumor-associated antigens (TAAs) [29, 30]. Figure 2 shows the mechanism of action of oncolytic viruses. According to the development, OVs are classified into two categories: naturally occurring OVs and genetically modifying OVs [31]. Natural OVs are wild-type viruses that selectively infect and replicate in tumor cells, leading to the lysis of infected cells. For example, reovirus only targets the epidermal growth factor receptor (EGFR) overexpressed cells and replicates in Ras‐activated tumor cells. EGFR produces a phospholipase that antagonizes double‐stranded RNA (dsRNA)‐dependent protein kinases by activating the Ras pathway, resulting in an enhancement of OV replication [32]. Despite the tumor-targeting properties of some viruses, immense interest has focused on other viruses for genetically modifying their genome to enhance their tropism toward tumor cells and boost immune responses. Among the various natural and genetically modified OVs, only talimogene laherparepvec (T-VEC), a modified herpes simplex virus type 1 (HSV-1), has been approved by the food and drug administration (FDA) for the treatment of patients with advanced melanoma in 2015 [33, 34]. Structurally, infected cell protein (ICP) 34.5 and ICP 47 genes were deleted in T-VEC and granulocyte–macrophage colony-stimulating factor (GM-CSF) was inserted into its genome [35]. ICP 34.5 gene is vital for infecting neurons and other healthy cells in HSV-1; thus, its deletion allows the virus replication within tumor cells which inhibits the protein kinase R (PKR) pathway and reduces neurotoxicity [36, 37]. ICP 47 gene prevents antigen loading of MHC I molecules through binding to transport associated protein, which results in a reduction in immune destruction of the virus-infected cells; thus, its deletion enhances MHC I expression and tumor antigen presentation in infected tumor cells [35, 38]. Moreover, the insertion of GM-CSF into the virus genome enhances the accumulation of dendritic cells (DCs) at inflammation sites and enhances antigen presentation function [39]. In addition to T-VEC, two other OVs have been approved for regional application: Oncorine (H101), an engineered adenovirus for head and neck cancer, in China and Rigvir, an unmodified picornavirus for melanoma, in Latvia, Armenia, and Georgia [40].

### Oncolytic virotherapy for head and neck cancer

Oncolytic viruses have been injected intratumorally (IT) and intravenously (IV) with excellent safety profiles in clinical trials. Various viruses have been used for the treatment of HNCs with both methods. Table 1 summarizes the application of multiple OVs for the treatment of HNCs in clinical trials and their combination with other therapeutic agents.

**Table 1:** Multiple OVs for the treatment of head and neck cancers in clinical trials

Virus family	Oncolytic agent	Phase	Administration route	Combination with	ClinicalTrials.gov ID
Measles virus	MV-NIS	I	IT	No	NCT01846091
HSV-1	HF10	I	IT	No	NCT01017185
Adenovirus	CAdVEC	I	IT	CAR-T cell	NCT03740256
Coxsackievirus 21	CAVATAK	I	IT	No	NCT00832559
Reovirus	Reolysin	II	IV	Paclitaxel/Carboplatin	NCT00753038
Reovirus	Reolysin	III	IV	Paclitaxel/Carboplatin	NCT01166542
HSV-1	ONCR-177	I	IT	Pembrolizumab	NCT04348916
Vaccinia virus	GL-ONC1	I	IV	Radiotherapy/Cisplatin	NCT01584284
VSV	VSV-IFNβ-NIS	I/II	IV	Pembrolizumab	NCT03647163
Adenovirus	VCN-01	I	IV	Durvalumab	NCT03799744
Adenovirus	OBP-301	II	IT	Pembrolizumab/SBRT	NCT04685499
Vaccinia virus	JX-594	I	IT	No	NCT00625456

IT: intratumoral; HSV-1: herpes simplex virus-1; IV: intravenous; VSV: vesicular stomatitis virus; SBRT: Stereotactic body radiation therapy

#### Oncolytic adenoviruses

Adenoviruses (Ads) and non-enveloped viruses containing ~ 36 kb double-stranded DNA (dsDNA) encode E1-E4 and L1-L5 as early and late genes, respectively [41]. Structurally, Ads capsid is composed of major proteins, including hexon, penton base, and fiber, and minor proteins, including IIIa, VI, VIII, and IX [42]. Different species of Ads have various receptors to infect the target cell. Coxsackievirus-Ad receptor (CAR) is known as the main receptor of Ads, while CD46, CD80, CD86, and desmoglein-2 (DSG2) are other receptors for Ads [43]. Ads require two interactions to infect target cells: (1) binding the fiber knob of the virus to the cell surface receptor and (2) binding the arginine-glycine-aspartic acid (RGD) motif in the penton base of Ads to target cells integrin, specifically αvß3 [44]. These interactions lead to viral endocytosis, the degradation of viral coat proteins, and the transcription of early and late genes in the nucleus of the host cell, resulting in Ad assembly and release from the cell.

The most prominent oncolytic adenovirus (OAd) is Oncorine, which was approved in China for treating nasopharyngeal carcinoma in combination with 5-fluorouracil (5-FU) and cisplatin chemotherapy in 2005 [45]. Oncorine is based on Ad vector serotype 5 (Ad5) in which viral E1B-55 k gene, as well as four regions in E3 gene, were deleted to guarantee its replication in p53-deficient tumor cells and its safety [46]. In addition to Oncorine, other OAds have been studied in clinical trials against HNCs, including CAdVEC, VCN-01, and OBP-301. CAdVEC is a binary construct composed of an OAd and helper-dependent Ad (HDAd) to increase cargo of transgene capacity of up to 34 kb besides the lytic activity [47]. Due to the resistance of HNSCC cells to chimeric antigen receptor T cell (CAR-T cell) therapy, the Suzuki team tested IL-12 and PD-L1-incorporated CAdVEC (CAdVEC*IL12*_*PDL1*) in combination with human epidermal growth factor receptor 2 (HER2)-specific CAR-T cells in a mice model. They found that CAdVEC*IL12*_*PDL1* could inhibit tumor growth and prolonge survival without losing body weight [48]. A phase I trial using CAdVEC mixed with HER2-specific CAR-T cells is under test for patients with HER2-positive solid tumors, including HNSCC (NCT03740256). VCN-01 is another OAd based on the Ad5 in which the genome is engineered to selectively replicate in pRB-deficient tumor cells, carry an integrin-binding motif RGD in the fiber shaft for targeting tumor cells, and express hyaluronidase for degrading extracellular matrix [49]. The efficacy and safety of VCN-01 in several tumor models, including HNC, have been demonstrated. Intratumorally (IT) administration of VCN-01 could reduce tumor growth and increase survival [49]. The combination of VCN-01 and Durvalumab is under phase I clinical trial for the treatment of recurrent/metastatic HNSCC (NCT03740256). Because of the high expression of the human telomerase reverse transcriptase (hTERT) in cancer cells [50], OBP-301 (Telomelysin) is an oncolytic virus based on the Ad5 in which promoter of the hTERT gene is inserted upstream of the E1 gene [51]. It has been shown that the effects of OBP-301 can enhance in combination with other therapeutic agents. For instance, Kondo et al*.* showed that a combination of OBP-301 with cisplatin had an additive anti-tumor effect on HNSCC when the chemotherapy agent preceded OBP-301 treatment [52]. Moreover, OBP-301 can overcome the resistance of HNCs to radiotherapy [53].

#### Oncolytic herpes simplex viruses

Herpes simplex viruses (HSVs) are enveloped viruses containing ~ 152 kb dsDNA, encoding about 80 proteins. Some advantages make HSVs an attractive candidate for oncolytic virotherapy: (1) Most of HSVs' genome is non-essential parts that provide the ability to manipulate and add transgenes [54], (2) HSVs are able to infect various cancer cells [55], and (3) targeting toward tumor cells by modifying their glycoprotein [56].

In addition to T-VEC, other oncolytic HSVs have been developed against various cancers. For instance, HF10 is a naturally mutated HSV without any deletion or insertion of transgenes with oncolytic activity. Esaki et al*.* indicated that HF10 could replicate in and kill HNSCC cells. They also showed that HF10 suppressed tumor growth and prolonged survival in an ear tumor model by inducing tumor necrosis with infiltration of CD8^+^ cells and releasing anti-tumor cytokines, including IL-2, IL-12, TNF-α, IFN-α, -β, and -γ [57]. ONCR-177 is a genetically engineered oncolytic HSV-1 carrying five transgenes: IL-12 for activation of natural killer (NK) and T-cells, CCL4 and the extracellular domain of FLT3LG for expansion and recruitment of DCs, and antagonists of PD-1 and CTLA-4 for overcome T-cell exhaustion. To reduce viral replication in normal cells and neuropathic activity as well as selectively targeting tumor cells, ONCR-177 also carries microRNA for the degradation of viral transcripts and is mutated in UL37 [58]. The combination of ONCR-177 and Pembrolizumab is under phase I clinical trial for the treatment of HNSCC patients (NCT04348916).

#### Other oncolytic viruses

In addition to Ads and HSVs, other oncolytic viruses have been used in clinical trials for treating HNCs. For instance, MV-NIS is an oncolytic virus in which the thyroidal sodium iodide symporter (NIS) is inserted into the measles virus (MV) genome to facilitate imaging virus-infected cells with single-photon emission computed tomography [59, 60]. To infect tumor cells, MV-NIS uses CD46 receptors on the cells and then fuses infected cells with un-infected neighbor ones, leading to the formation of multinucleated syncytia [59]. A phase I trial using IT administration of MV-NIS is under test for patients with HNSCC (NCT01846091). The other oncolytic virus under clinical trial is CAVATAK (NCT00832559). CAVATAK (Coxsackievirus A21, CVA21) infects tumor cells via binding to the intracellular adhesion molecule-1 (ICAM-1), which is highly expressed on various cancer cells, including HNCs [61, 62]. Reolysin (Pelareorep) is a natural oncolytic virus derived from human Reovirus Serotype3-Dearing Strain containing cytotoxic effects on tumor cells with an activated Ras pathway [63, 64]. Owing to the safety and tolerability of intravenous (IV) administration of Reolysin in combination with Paclitaxel/Carboplatin in HNSCC patients [65], phase II and III clinical trials of the combinational regimen have been conducted (NCT00753038 and NCT01166542). Vaccinia virus has been attracted attention as another valuable oncolytic virus because it can target various cells, replicate in the cytoplasm, and carry large transgenes [66]. GL-ONC1 (GLV-1h68) is an oncolytic virus based on the vaccina virus in which viral thymidine kinase (TK), hemagglutinin (HA), and F145L genes are replaced with β-galactosidase, β-glucuronidase, and Renilla luciferase/green fluorescence (RLuc-GFP), respectively [67]. In a phase I clinical trial, Mell et al*.* found that IV administration of GL-ONC1 combined with chemotherapy and radiotherapy in patients with HNCs could enhance overall and progression-free survival [68]. JX-594 (Pexa-Vec) is an oncolytic vaccinia virus with three modifications on its genome: TK deletion, lac-Z gene insertion under the control of p7.5 promoter, and GM-CSF gene insertion [69]. The safety of JX-594 is under investigation for solid tumors, including HNCs (NCT00625456).

### Challenges of oncolytic virotherapy

Similar to other therapeutic strategies, the application of OVs also has challenges and scientists are trying to overcome these challenges to reach an optimum and ideal candidate in cancer therapy. One of the most important challenges is pre-existing immunity against viruses due to previous infection or immunization, reducing OVs' efficiency [70–72]. Several approaches have been developed to minimize the unpleasant impact of pre-existing immunity, including coating OVs with polymers, using cellular carriers, and using immunosuppressive drugs such as cyclophosphamide [73]. For example, shielding OAds with polymers not only increases their half-life in blood but also reduces Ads immunogenicity and hepatotoxicity. Moreover, modifications of polymers can be applied to target the virus toward specific tumor cells [74]. Doronin et al*.* found that PEGlyated OAd with a 20-kDa PEG reduced liver uptake compared with naked OAd or 5-kDa PEGylated OAd up to 19 or 90 fold, respectively. Furthermore, the survival in xenograft-bearing mice was increased from 14 to 31 days in naked OAd-treated mice compared to 20-kDa PEGylated OAd- treated mice [75]. The pre-existing immunity obstructs the systemic delivery (IV administration) of viral particles to the tumor cells, limiting delivery routes to IT injection [76]. Recently, some studies revealed that pre-existing immunity could potentiate anti-tumor activity of OVs [77, 78]. Ricca et al*.* investigated the role of pre-existing immunity against oncolytic Newcastle Disease Virus (NDV) on its therapeutic efficacy in mice models. They indicated that while pre-existing immunity limited NDV replication in tumor cells, abscopal anti-tumor immune effects, tumor growth, and survival were not compromised; on the contrary, they were superior, suggesting pre-existing immunity may increase NDV therapeutic efficacy by enhancing systemic immune responses [78].

Intercellular junctions, specifically tight junctions, act as barriers against virus penetration, leading to resistance to OVs. Genetically modified OVs can open the junctions by producing proteins such as junction openers (JOs). Yumul et al*.* reported that a JO-expressing OAd had a more remarkable anti-tumor effect than an unmodified OAd [79]. The positive anti-tumor effect of JO also may be related to its ability to unmask tumor-related antigens decoyed in epithelial junctions [80]. Using extracellular matrix (ECM) degrading enzymes is another strategy in enhancing the spreading and penetration of OVs. For example, co- or pre-treatment of tumors with ECM degrading enzymes, collagenase or hyaluronidase, enhanced the spread of oncolytic HSVs and Ads and their therapeutic effects [81, 82]. In another study, Mok et al*.* found that overexpression of matrix metalloproteinases (MMPs), MMP-1 and MMP-8, by tumor cells could increase an oncolytic HSV distribution and improve their efficacy [83]. Besides JO proteins and ECM degrading enzymes, fusogenic proteins, including Gibbon-Ape Leukemia Virus fusogenic membrane glycoprotein and fusion-associated small transmembrane (FAST) proteins, have attracted attention for enhancing the IT spread of OVs [84]. After infecting tumor cells, the IT distribution of fusogenic protein-expressing viruses is facilitated owing to the fusion between the infected and neighboring cells [85]. Fusogenic proteins forms not only nonviable multinuclear cells but also release minimal mature virion into the blood circulation and healthy tissues [86]. Moreover, using this strategy reduces the need for the administration of the virus at high titer due to a single virion's ability to infect neighbor cells [87].

Certainly, using combinational strategies in which OVs are combined with other therapeutic agents, including chemotherapy and CAR-T cells, can be applied to improve the efficacy of OVs. In the combination of OVs and CAR-T cells, OVs can generate a chemotactic TME for CAR-T cells. In addition, redirecting immune responses against OVs toward the tumor will improve the anti-tumor effects of therapeutic viruses. Furthermore, facilitating the immune tolerance of viral antigens by some strategies, including infusing tolerogenic DCs and systematic administration of dominant viral antigens, may modulate immune responses against the virus.

## Monoclonal antibodies

### Overview of monoclonal antibodies

**Fig. 3:**
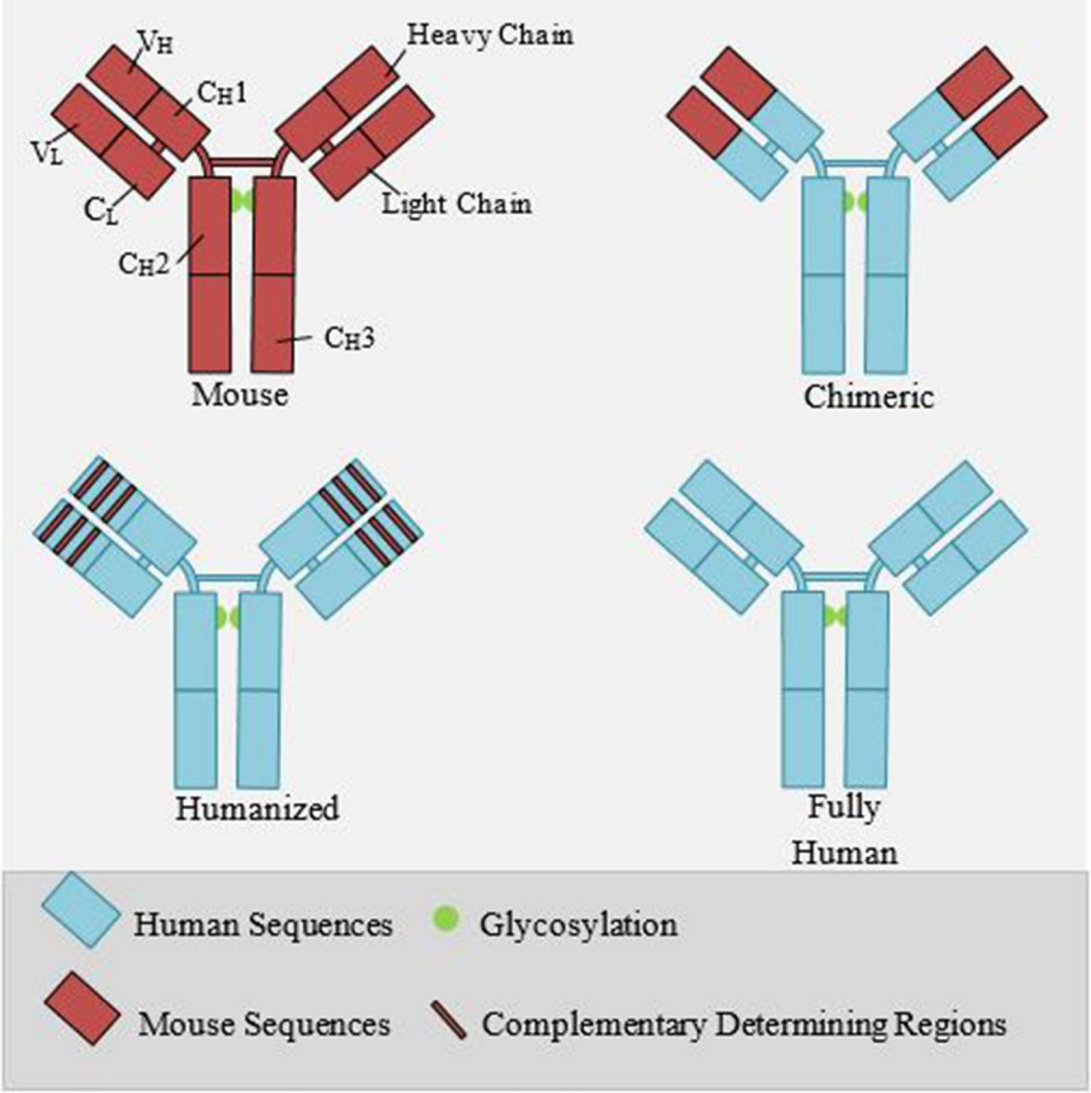
The structure of monoclonal antibodies. In the murine mAbs, all the Fab and Fc regions are derived from mice, whereas only variable domains and complementarity-determining regions (CDRs) in chimeric and humanized mAbs, respectively, have mice origin. Human mAbs are fully human

As the body's defense mechanism, B lymphocytes produce antibodies against foreign substances, called antigens, to recognize regions on the antigens (epitopes) and mark them for destruction with other immune cells. Monoclonal antibodies (mAbs) are produced by unique B cell clones that bind to specific epitopes. The mAb technology was discovered by Köhler and Milstein in 1975 using the hybridoma technique in which cells from the spleen of an immunized rat were fused with a mouse myeloma cell line [88]. The immunogenic and allergic issues of mouse-derived mAbs in humans led to invent of chimeric and humanized antibodies to overcome these limitations. Chimeric mAbs are made with a fusion of variable regions from a mouse with the constant regions from a human [89]. In humanized mAbs, only the hypervariable regions (CDRs) stem from mice and the other regions originate from humans [90]. The state-of-the-art of mAbs are fully human ones are developed by phage display technology or transgenic mice [91]. Figure 3 represents the structure of different mAbs.

Over the past decades, the importance of diagnostic and therapeutic mAbs has been intensely increased because they have multiple disease targets. Five of ten best-selling innovative drugs were mAbs and one was Fc-fusion protein in 2016, while 61 and 11 mAbs and Fc-fusion proteins, respectively, were in the market [92]. The FDA-approved therapeutic mAbs were reached 79 mAbs in 2019, including 30 mAbs for the treatment of various cancers [93]. In cancer immunotherapy, Rituximab, a chimeric mAb that targets CD20, is the first FDA-approved mAb for B-cell non-Hodgkin’s lymphomas immunotherapy in 1997 [94, 95]. In the next section, we will review mAbs and their targets in the immunotherapy of HNCs.

### Monoclonal antibodies for head and neck cancer

#### Targeting angiogenesis

Solid tumors need new blood vessels that result from the sprouting of previous ones to provide nutrients and oxygen for further growth, a process called angiogenesis [96]. There are various stimulators of angiogenesis, including fibroblast growth factor 2 (FGF2), transforming growth factor‐β (TGF‐β), epidermal growth factor receptor (EGFR), interleukin-8 (IL-8), angiopoietins, hepatocyte growth factor (HGF), and vascular endothelial growth factor (VEGF) [97, 98]. The VEGF family, consisting of placental growth factor (PlGF) and VEGFA-E, is a crucial regulator of angiogenesis that exerts its angiogenic effect by binding to VEGF receptors VEGFR1-3 [99]. Table 2 summarizes various mAbs and their application in the treatment of HNCs in clinal trials.

**Table 2:** Monoclonal antibodies in clinical trials for the treatment of head and neck cancers

mAb	Target	Phase	Combined with	ClinicalTrials.gov Identifier
Bevacizumab	VEGF-A	III	Chemotherapy	NCT00588770
Bevacizumab	VEGF-A	I/II	Erlotinib	NCT00055913
Ramucirumab	VEGFR2	I/II	Pembrolizumab	NCT03650764
Ficlatuzumab	HGF	II	Cetuximab	NCT03422536
Cetuximab	EGFR	II	–	NCT03769311
Cetuximab	EGFR	II	Afatinib	NCT02979977
Trastuzumab	HER2	II	–	NCT00004163
Panitumumab	EGFR	II	Paclitaxel	NCT01264328
Panitumumab	EGFR	I	Chemotherapy	NCT00513383
Nivolumab	PD-1	II	Paclitaxel	NCT04282109
Nivolumab	PD-1	II	Relatlimab/Ipilimumab	NCT04080804
Pembrolizumab	PD-1	I	Clopidogrel/Acetylsalicylic acid	NCT03245489
Pembrolizumab	PD-1	II	Tadalafil	NCT03993353
Atezolizumab	PD-L1	II	Bevacizumab	NCT03818061
Durvalumab	PD-L1	II	Cetuximab	NCT03691714
Durvalumab	PD-L1	II	Carboplatin/Paclitaxel	NCT03723967
Ipilimumab	CTLA-4	I	–	NCT02812524
Tremelimumab	CTLA-4	II	Durvalumab/Radiotherapy	NCT03624231

Bevacizumab, an anti-VEGFA mAb, was approved for colorectal cancer treatment in 2004 and has been approved for glioblastoma, non-small-cell lung carcinoma (NSCLC), kidney, cervical, and ovarian cancers to date [100]. Several studies investigated the effect of bevacizumab in combination with different chemotherapeutic agents and radiotherapy in HNCs [101–103]. In phase III clinical trial, Argiris et al*.* reported that although combining bevacizumab with chemotherapy could improve progression-free survival (6.0 months versus 4.3 months, *P* = 0.0014) and response rate (35.5% versus 24.5%, *P* = 0.016) without improving OS compared with chemotherapy regimen, this combinational regimen increased toxicity, including bleeding events (6.7% versus 0.5%, *P* < 0.001) and treatment-related deaths (9.3% versus 3.5%, *P* = 0.022) [104]. Ramucirumab is another anti-angiogenesis mAb that targets VEGFR2. Spratlin et al*.* found that ramucirumab had a satisfactory therapeutic effect in treating solid tumors, such as HNC [105]. Due to the impairment of hepatocyte growth factor (HGF)/cMet pathway in resistance to EGFR-targeted therapy, Bauman et al*.* investigated the therapeutic activity of a combination of anti-HGF mAb, ficlatuzumab, and anti-EGFR mAb, cetuximab for treating recurrent/metastatic HNC patients. They showed that the combinational regimen had a manageable safety profile with promising anti-tumor activities, such as increased CD8^+^ T-cells, with 8.9 months OS and 5.4 months PFS [106]. In a phase III clinical trial, Bonner et al*.* demonstrated that cetuximab in combination with radiotherapy remarkably improved OS at 5-year. The 5-year OS in the cetuximab-plus-radiotherapy group and the radiotherapy-alone group were 45·6% and 36·4%, respectively [107].

#### Immune checkpoint inhibitors

In response to antigens and activation, T-cells require two interactions: Binding of T-cell receptors to antigens on MHC-I and binding of co-stimulatory (co-inhibitory) receptors of T-cells to co-stimulatory (co-inhibitory) ligands [108]. Several co-inhibitory receptors have been identified in cancer, including PD-1, CTLA-4, T-cell immunoglobulin and mucin domain 3 (Tim-3), lymphocyte-associated gene 3 (LAG3), B and T lymphocyte attenuator (BTLA), and T-cell immunoglobulin and ITIM domain (TIGIT), called immune checkpoints [109]. mAbs blocking immune checkpoints, particularly PD-1 (or PD-L1 as its ligand) and CTLA-4, have attracted attention due to their impressive results in clinical trials compared with chemotherapy and other conventional therapeutic agents.

For targeting and blocking PD1 and PD-L1, four mAbs have been approved for cancer therapy: pembrolizumab and nivolumab against PD-1, atezolizumab and avelumab against PD-L1. Pembrolizumab and nivolumab have been approved for recurrent and metastatic HNSCC patients based on the results of KEYNOTE 012 and CHECKMATE 141 clinical trials [110]. A phase III clinical trial, KEYNOTE 048, showed promising anti-tumor effects of pembrolizumab in treating relapsed or metastatic HNSCC patients. The OS of pembrolizumab plus chemotherapy patients was 13.0 months compared to patients who received cetuximab plus chemotherapy with 10.7 months (*P* = 0.003 4) [111]. According to the CHECKMATE 141 trial, the median survival of patients treated with nivolumab (3 mg kg^−1^) and the standard treatment (cetuximab, docetaxel, or methotrexate) were 7.5 and 5.1 months, respectively (95% confidence interval, *P* = 0.01). In addition, severe toxic effects in the nivolumab-treated group were 13.1% compared with the standard treatment group with 35.1%. The most frequent adverse effects in the nivolumab group were nausea, fatigue, decreased appetite, pruritus, and rash. In addition, gastrointestinal and skin events were less and more common adverse effects in the nivolumab group compared to the standard group [112, 113].

Because of binding to B7, CTLA-4 can prevent the interaction between the co-stimulatory molecule CD28 and B7, results in restricting T-cell proliferation and IL-2 production [114]. Currently, anti-CTLA-4 mAbs, including ipilimumab and tremelimumab, are under phase III clinical trials against various cancers. In Phase II, the CONDOR clinical trial revealed that the combination of tremelimumab and durvalumab in HNSCC patients with low or no PD-L1 expression had a manageable toxicity profile and minimal differences in clinical benefits compared with durvalumab monotherapy [115]. Similarly, a phase III study demonstrated that durvalumab or tremelimumab plus durvalumab did not significantly increase OS compared with standard of care [116]. As first-line treatment for recurrent or metastatic HNSCC, the safety and efficacy of ipilimumab plus nivolumab are under clinical trials (NCT02741570 and NCT02823574).

### Challenges of monoclonal antibodies

Despite remarkable achievements and advances in using and developing mAbs, there are some challenges in their clinical uses. One of the most substantial challenges in applying immune checkpoint inhibitors is the selection of appropriate patients and the lack of reliable predictive biomarkers for anticipating response rates to therapeutic mAbs [117]. Although the expression of PD-L1 is considered a biomarker in response to mAbs against PD-1 and PD-L1, there are some variabilities between clinical trials [118, 119]. The inconsistency could be due to sampling error, intratumoral heterogeneity, low or no standardization of PD-L1 assays and using different mAbs in the assays, and different scoring and cut-offs for determining the positivity of PD-L1 expression [117]. A combination of other predictive biomarkers, specifically non-invasive biomarkers presented in liquid biopsies, may enhance the ability to detecting patients with the highest benefit from mAbs.

There are some technological challenges in producing mAbs. Inappropriate formulation and preparation of mAbs can lead to oxidation, deamidation, isomerization, fragmentation, denaturation, and aggregation [120]. Modifications of crucial residues can affect (pre)clinical development and the success rate of mAbs. For instance, tryptophan (W) amino acid can enhance immunogenicity and reduce the solubility of the product by inducing aggregation [121]. As the standard purification method of mAbs, Protein A affinity chromatography has gained great interest due to its high purity levels and binding affinity. However, the limited lifetime of resins and their elevated costs, as well as caustic instability and ligand leaching, are the main concerns of this technology. To increase their lifetime, stationary phases' ability to tolerate high concentrations of NaOH can improve resin cleaning and disinfection. Moreover, Protein A combined with novel affinity ligands or using novel affinity ligands have been applied for effective mAb separation due to the disability of Protein A ligands in binding to all types of IgG [122].

The other important issue in the clinical application of mAbs is delivery routes due to their biological and physicochemical properties, including poor stability and large molecular weight, which make them susceptible to gastrointestinal proteases and hinder their transport through biological membranes; thus, oral delivery is not suitable for mAb administration. The high cost of administration because of the need for hospital administration and pain for patients are drawbacks of IV administration of mAbs [123]. The subcutaneous (SC) route is currently preferred to others due to less invasiveness, the ability of self-administration, and no requirement for medical personnel [120].

## CAR-T cell therapy

### Overview of CAR-T cell therapy

Chimeric antigen receptor (CAR)-T cell therapy defines genetic modification and redirection of the patient's T-cell to target tumor antigens and kill cancer cells. CAR-T cell therapy has achieved significant successes against hematological malignancies because of two USA FDA-approved CAR-T cells targeting CD-19: KYMRIAH (Tisagenlecleucel) from the Novartis (East Hanover, NJ USA) for treatment of B-cell acute lymphoblastic leukemia (B-ALL) and YESCARTA (Axicabtagene ciloleucel) from the Kite Pharma (Santa Monica, CA USA) for treating diffuse large B-cell lymphoma (DLBCL) [124]. To construct CAR-T cells, patient PBMCs are collected, T-cells are isolated and activated, and then a CAR-encoding cassette is transduced into T-cells to express a specific receptor. Finally, the generated CAR-T cells are infused into patients' blood after expansion in vitro [125].

**Fig. 4:**
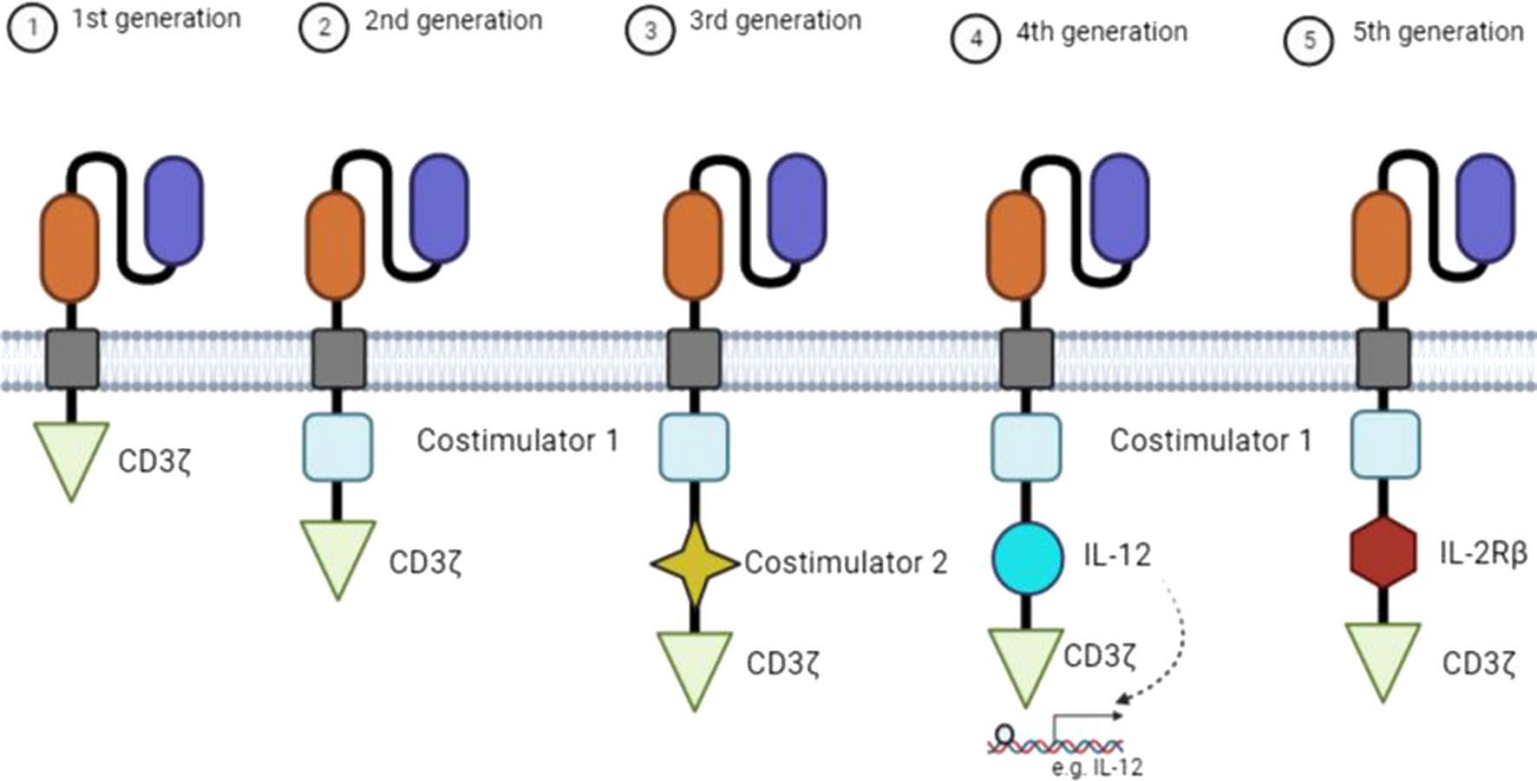
Five generations of CAR-T cells. First-generation CAR-T cells include the CD3ζ alone as the intracellular domain, whereas the second-generation CARs consist of additional costimulatory intracellular domains, such as CD28 or 4-1BB (CD137). Third-generation CARs consist of two costimulatory intracellular domains, such as CD28 and 4-1BB. The fourth- and fifth-generation CAR-T cells are based on the second-generation ones. The fourth-generation CARs can induce the expression of cytokines, such as IL-12, whereas the fifth-generation CAR-T cells include an intracellular domain of cytokine receptors, such as IL-2Rβ

Structurally, a CAR-T cell contains four components: a single-chain variable fragment (scFv) domain derived from antibodies for recognizing and binding to tumor-associated antigens (TAAs), a hing domain that provides flexibility and access to TAAs, a transmembrane (TM) domain for influencing the expression and stability of the receptor, and an intracellular domain that mediates signal transduction [126]. According to the intracellular domain structure and composition, five generations of CAR-T cells have been created (Fig. 4). The first-generation CAR-T cells contain a CD3ζ chain for signal transduction [127]. Due to limited anti-tumor activities of the first-generation CAR-T cells and a decrease in their proliferation [128], a co-stimulatory molecule, such as 4-1BB receptor (CD137) or CD28, is incorporated in the second-generation CAR-T cells. The third-generation CARs carry two co-stimulatory molecules. The fourth- and fifth-generation CARs are designed based on the second-generation CAR-T cells. In the fourth-generation CARs, the intracellular domain contains additional domains for the secretion of a cytokine. The fifth-generation CAR-T cells, also known as next-generation CARs, carry intracellular domains of cytokine receptors, such as IL-2Rβ, with a binding site for the STAT3 transcription factor [129].

### CAR-T cell therapy for head and neck cancer

For HNCs, several CAR-T cells against different antigens have been developed and translated into clinical trials (Table 3).

**Table 3:** CAR-T cell therapy in clinical trials for head and neck cancers

Target	Phase	Sponsor	ClinicalTrials.gov Identifier
ErbB	I/II	King's College London	NCT01818323
HER2	I	Baylor College of Medicine	NCT03740256
EpCAM	I	Sichuan University	NCT02915445
NKG2D	I	CytoMed Therapeutics Pte Ltd	NCT04107142
LMP1	I/II	The Second Hospital of Nanjing Medical University	NCT02980315
LMP1, LMP2, and EBNA1	II	Fujian Cancer Hospital	NCT03648697

One of the attractive antigens in CAR-T cell therapy of HNCs is the ErbB family that belongs to receptor tyrosine kinases (RTKs) and comprises four members, including ErbB-1 (EGFR), ErbB-2 (HER2/neu), ErbB-3, and ErbB-4. It has been shown that ErbB signaling is dysregulated in HNSCC. For example, EGFR is upregulated in more than 90% of cases and its upregulation is correlated with radioresistance, metastasis, and poor survival [130]. For this reason, T4-immunotherapy has been developed in which T-cells are engineered to co-express T1E28ζ and 4αβ. The T1E28ζ includes the promiscuous ErbB ligand, T1E, CD3ζ, and CD28 [131]. T1E can bind to several ErbB hetero- and homodimers, eliciting broad anti-tumor activities [132]. Moreover, the 4αβ receptor in which L-4 receptor α extracellular domain is fused to the TM and intracellular domain of the IL-2/15 receptor β chain enables the selective enrichment of CAR-T cells during manufacture [133]. Currently, the safety of T4 immunotherapy is under investigation in patients with HNCs (NCT01818323). A pre-clinical study revealed that combining two immunotherapy strategies, oncolytic virotherapy carrying checkpoint inhibitor and CAR-T cell therapy, significantly enhances survival and controls primary and metastasized tumors [48]. Thus, combining different immunotherapy strategies can be considered a promising therapeutic approach in treating HNCs.

### Challenges of CAR-T cell therapy

Potentially severe toxicities limit CAR-T cell therapy and cytokine release syndrome (CRS) is the most common type of CARs-related toxicity. Cytokine production, specifically inflammatory cytokines, by infused CARs or other immune cells in response to produced cytokines by CARs can lead to CRS, characterized by hypotension, high fevers, hypoxia, and multi-organ dysfunction [134]. It has been shown that the structure of CAR-T cells is a determinant factor in CRS. For example, CRS begins earlier in response to CARs with a CD28 co-stimulatory domain than CARs containing a 4–1BB co-stimulatory domain [135]. Neurological toxicities are another drawback of CAR-T cell therapy, characterized by encephalopathy, cognitive disorders, headache, aphasia, tremor, and focal weakness [136, 137]. The binding of CAR-T cells to antigens also expressed on non-tumor cells known as on-target/off-tumor toxicity, leading to the destruction of normal cells and tissues. Thus, antigen specificity is a crucial factor in designing CAR-T cells. Several strategies have been developed to reduce off-target toxicity, such as utilizing dual antigen targeting CARs and controlling the expression and activity of CAR through incorporating a switch element [138, 139].

The necessary prerequisite in CAR-T cell therapy for efficiently working is the access of CARs to target antigens on the tumor cells and their infiltration into the tumor microenvironment. ECM and its components are the main physical barriers for CAR-T cells. To increase their infiltration, Caruana et al*.* engineered T-cells to construct heparanase expressing CAR-T cells. They showed an improvement in tumor infiltration and anti-tumor activity of the constructed CARs [140]. Zhang et al*.* exerted macrophages' ability to produce MMPs for remodeling ECM and improve CAR-T cells infiltration into tumors [141]. Since cancer-associated fibroblasts (CAFs) form a large mass of tumors and act as a barrier for entering and activity of therapeutic agents as well as their paracrine and protumoral effects on tumor cells [142], CAFs targeting CARs could be an attractive approach. Wang et al*.* indicated that fibroblast activation protein (FAP)-targeting CARs could inhibit tumor growth by reducing FAP(hi) stromal cells [143]. In addition to infiltration obstacles, the migration of CAR-T cells also is hindered from trafficking toward tumor sites. To enhance the localization of CARs to tumors, modulation of cytokines has been explored. For instance, Craddock et al*.* indicated that the expression of CCR2b, the receptor of CCL2 chemokine that is upregulated by tumor cells, could enhance the trafficking of anti-GD2 CAR-T cells in the tumor microenvironment [144].

The immunosuppressive tumor microenvironment with immunosuppressive cells, including Tregs, TAMs, and MDSCs, is another factor that hinders the optimal efficacy of CAR-T cells in solid tumors. Burga et al*.* found that the expansion of MDSCs in a GM-CSF-dependent manner and their PD-L1 expression could suppress CAR-T cells' anti-tumor effects by engaging PD-1 on CARs. They also indicated that blocking GM-CSF with CARs was a potential approach for enhancing the therapeutic efficacy of CAR-T cells [145]. Combining CAR-T cells with immune checkpoint inhibitors provides a synergic anti-tumor activity owing to increased PD-1 expression on CAR-T cells during the time. Cherkassky et al*.* revealed that blocking the PD-1/PD-L1 pathway using an anti-PD-1 antibody, PD-1 shRNA, or a PD-1 dominant negative receptor could restore the cytotoxic function of CD28 CAR-T cells [146].

## Vaccines

### Overview of cancer vaccines

**Fig. 5:**
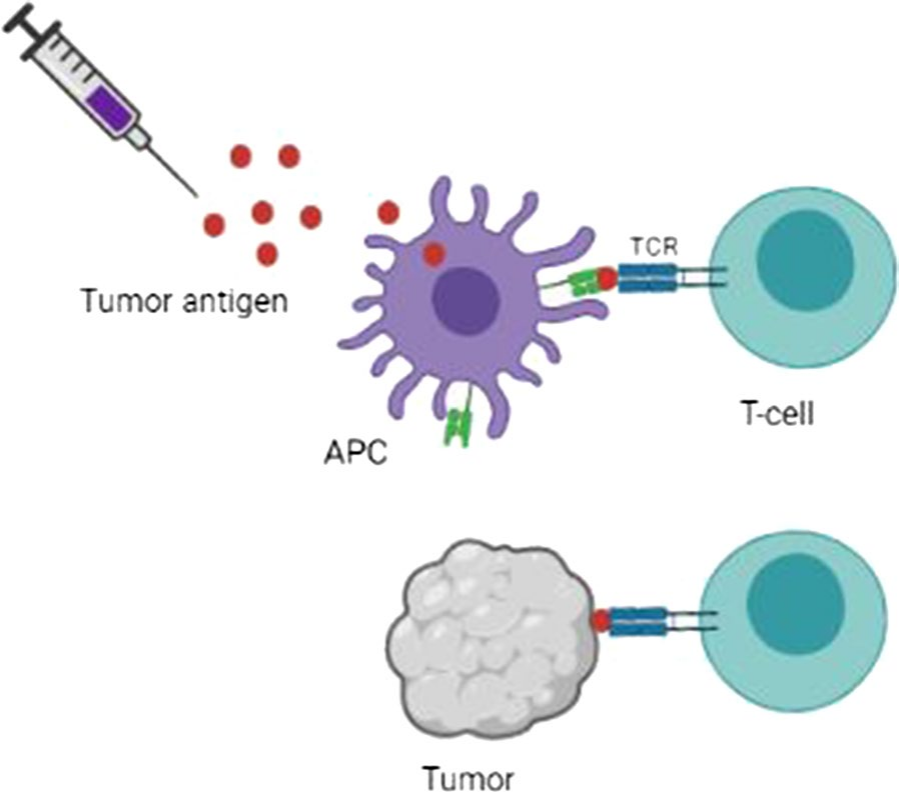
Therapeutic vaccines against cancer. Following the administration, the tumor antigens are uptaken and processed by antigen-presenting cells (APCs) to present on major histocompatibility complex (MHC) classes to T-cells and activate them against the antigen

Although three prophylactic vaccines, including Cervarix, Gardasil®, and Gardasil®9, have been approved against HPV-induced diseases and cancers, they are not effective in treating pre-existing ones; thus, targeting antigens expressed in the tumor by therapeutic vaccination is crucial. For HPV-positive malignancies, viral antigens have been considered, while TAAs and tumor-specific antigens (TSAs) are available for HPV-negative cancers [147]. Various vaccine platforms have been developed to deliver antigens or epitopes, including DNA/mRNA vaccines, peptide vaccines, viral/bacterial-based vaccines, and cellular vaccines. Figure 5 represents the mechanism of action of therapeutic vaccines. Stability, flexibility, safety, and simplicity of preparation are advantages of DNA vaccines, whereas they are low immunogen in patients [148]. Vaccines based on mRNAs are becoming gradually attractive owing to safety, high potency, rapid development, and low-cost preparation, whilst their inefficient delivery in in vivo and instability restricts the application of mRNA vaccines [149]. To maintain the activity of mRNA-based vaccines, various strategies have been applied, including freeze-drying [150], spray-drying, lyospher generation, and forming lipid nanoparticles (LNPs) [151]. Due to the FDA approval of mRNA-based vaccines from Moderna and Pfizer-BioNTech against the coronavirus disease 2019 (COVID-19), mRNA-based vaccines have attracted extensive interest in both infectious disease and cancer applications [152]. Using next-generation sequencing (NGS) and identifying mutations of cancer cells in patients, neoepitopes are constructed in mRNA form and are used as personalized cancer vaccines for eliciting immune responses. However, there are some challenges in developing mRNA-based vaccines against cancer compared to infectious diseases such as COVID-19. First, the vaccines of most infectious diseases are prophylactic, whereas cancers require therapeutic vaccines. Second, needing repeatable/multiple dosing of therapeutic vaccines compared with prophylactic ones raise the safety concern of mRNA-based vaccines [153]. Although peptide-based vaccines are safe and easy to produce, they need adjuvants to induce strong immune responses [147]. In vaccines based on viral vectors, attenuated viruses are applied to deliver target antigens in the infected cells, whereas in cellular vaccines, various cells have been utilized, such as tumor cells and DCs.

### Vaccines for head and neck cancer

Vaccines targeting viral antigens, specifically E6 and E7 oncoproteins, have demonstrated efficacy in HPV-positive HNCs and several clinical trials are being conducted to assess their safety and effectiveness. For instance, a phase 1b/2a clinical trial is evaluating the safety and efficacy of a DNA vaccine contains three plasmids expressing HPV16/18 E6 and E7 proteins with IL-12 (called MEDI0457) in combination with durvalumab (NCT03162224). ADXS11-001 (ADXS-HPV), a live attenuated Listeria monocytogenes (Lm)-LLO targeting HPV16 E7, is another therapeutic vaccine against the viral antigen (NCT02002182). TG4001 and ISA101 vaccines also are under clinical trial against HPV antigens in combination with checkpoint inhibitors (NCT03260023 and NCT02426892).

In addition to viral antigens, TAAs and TSAs are promising targets in designing therapeutic vaccines in HPV-negative HNCs, including mucin 1 (MUC1), carcinoembryonic antigen (CEA), and human telomerase reverse transcriptase (hTERT). MUC1 is a TM glycoprotein on the surface of nearly all epithelial cells that its aberrant expression is associated with malignant phenotype; thus, it is an ideal target for developing cancer vaccines [154]. A MUC1 vaccine combined with Tadalafil is under phase I clinical trial to treat HNCs (NCT02544880). The trialists believed that lowering MDSCs and Tregs by Tadalafil can prime a permissive environment and anti-tumor immune response to increase the efficacy of the MUC1 vaccine. Although the expression of CEA, a glycoprotein with cell–cell adhesion function, begins in gastrointestinal tissues during fetal development and continues throughout life, it is upregulated in epithelial cancers, such as HNCs [155, 156]. It has been shown that the GI-6207 vaccine (recombinant Sarrcharomyces Cerevusua-CEA) could downregulate Tregs and increase CD4^+^ and CD8^+^ T-cells in some patients [157]. Telomeres are located at the ends of each chromosome consist of repetitive hexanucleotide DNA sequences (TTAGGG), which their length is shorten with each cell replication, whereas its higher expression is found in different cancer types [158]. Two therapeutic vaccines, INO-1400 and UCPVax, have targeted hTERT in patients with HNCs in combination with IL-12 DNA and atezolizumab, respectively (NCT02960594 and NCT03946358).

Because of the genetic instability of cancer cells, a large number of mutations lead to the production of tumor-specific antigens, which are stated as neoantigens [159]. Neoantigens are extremely immunogenic due to their absence in normal tissues and can induce CD4^+^ and CD8^+^ T-cells to generate a robust immune response; thus, they have great potential to become new immunotherapy targets [160]. Currently, a neoantigen vaccine called GEN-009 in combination with two immune checkpoint inhibitors, pembrolizumab and nivolumab, is being conducted against several solid tumors, such as HNSCC (NCT03633110).

### Challenges of cancer vaccines

The development of vaccines against cancers faces unique challenges that generally do not hinder vaccines' development for infectious diseases. Therapeutic vaccines are designed to engage and induce tumor-specific T-cells, both naïve and dormant T-cells, which require antigens and adjuvants for stimulating APCs and, finally, optimal T-cell activation. Compared to other immunotherapy strategies, vaccines have been less remarkable due to improper selection of vaccine platform or adjuvant, suboptimal antigen selection, and unsuitable route/mode of delivery [161]. Moreover, cancer immune evasion, both intrinsic and extrinsic, is another obstacle in using vaccines. Defects in MHC I proteins [162], infiltration of suppressive cells such as MDSCs [163], and secretion of immunosuppressive cytokines and chemokines such as IL-10, TGF-β, and indoleamine 2,3-dioxygenase (IDO) [164] are the main mechanisms of cancer immune evasion that reduce the effectiveness of cancer vaccines. Therefore, combining vaccines with other therapeutic strategies to target immune-suppressive cells, inhibit immune checkpoint activation, and stimulate infiltration of local immune cells will maximize their clinical benefit. To fight immune-suppressive cells and subsequently mediators, decreasing the numbers or depleting Tregs and MDSCs in cancer patients will be useful strategies. In addition, immune checkpoint inhibitors can augment vaccine responses by eliminating the suppressive function of Tregs and MDSCs. Also, using adjuvants such as CpG oligodeoxynucleotides (CpG ODN), a ligand for toll-like receptor 9 (TLR9), together with vaccines can restore impaired immune responses. One of the obstacles in the infiltration of local immune cells is impaired vascular with poor adhesion molecules for leukocytes. Certainly, restoring the expression of endothelial adhesion molecules and promoting leukocyte-endothelium interactions can potentiate infiltration of immune cells into TME.

## Conclusions

During recent decades and based on our understanding of cancer immunology, attention to cancer immunotherapy has dramatically increased. Many challenges are still unsolved for cancer immunotherapies in a variety of malignancies. The understanding of the immune situation and the strength of immune responses in the tumor microenvironment is critical for designing and applying an immunotherapy strategy against cancer. In addition to selecting an appropriate therapeutic strategy, identifying predictive biomarkers for patient selection and predicting the clinical response is crucial in cancer immunotherapy. Furthermore, the combination of different strategies due to the heterogeneity of the tumor microenvironment will improve the therapeutic efficacy of cancer immunotherapy.

## Data Availability

Not applicable.

## Abbreviations

HNCs: Head and neck cancers
HNSCCs: Head and neck squamous cell carcinomas
HPVs: Human papillomaviruses
Treg: Regulatory T-cells
PD-1: Programmed cell death protein 1
CTLA-4: Cytotoxic T-lymphocyte-associated protein 4
TME: Tumor microenvironment
MDSCs: Myeloid-derived suppressor cells
OS: Overall survival
OVs: Oncolytic viruses
OAd: Oncolytic adenovirus
HSVs: Herpes simplex viruses
mAbs: Monoclonal antibodies
CAR-T cells: Chimeric antigen receptor-T cells
TAAs: Tumor-associated antigens
